# *In Vitro* and* In Vivo* Immunomodulator Activities of* Allium sativum *L.

**DOI:** 10.1155/2018/4984659

**Published:** 2018-06-12

**Authors:** Mouna Moutia, Norddine Habti, Abdallah Badou

**Affiliations:** ^1^Laboratory of Hematology and Cellular and Genetic Engineering, Faculty of Medicine and Pharmacy, Hassan II University, Casablanca, Morocco; ^2^Laboratory of Experimental Medicine and Biotechnology, Faculty of Medicine and Pharmacy, Hassan II University, Casablanca, Morocco; ^3^Cellular and Molecular Pathology Laboratory, Faculty of Medicine and Pharmacy, Hassan II University, 19 Rue Tarik Ibnou Ziad, B.P. 9154 Casablanca, Morocco

## Abstract

*Allium Sativum *L. (garlic), which is a species of the onion family, Alliaceae, is one of the most used plants in traditional medicine worldwide. More than 200 chemicals with diverse properties have been found in garlic extracts. Several garlic compounds were suggested to be efficient in improving various pathologies including certain types of cancer. This paper is an overview of data about garlic biological activities* in vitro* and/or* in vivo* on immune cells, on the development of certain inflammatory diseases, and on different types of carcinomas and sarcomas. Garlic and its compounds were found to have notable antioxidant properties. Garlic therapeutic potential has also been studied in several inflammatory diseases such as allergic-airway inflammation, inflammatory bowel disease, arthritic rheumatism, and atherosclerosis. Furthermore, garlic was found to be able to maintain the immune system homeostasis and to exhibit beneficial effects on immune cells especially through regulation of proliferation and cytokine gene expression. Finally, we will show how major garlic components such as sulfur compounds and polyphenols might be responsible for the garlic biological activities revealed in different situations. If identified, specific compounds present in garlic could potentially be used in therapy.

## 1. Introduction

*Allium Sativum *L. (Garlic), which is a species of the onion family, Alliaceae, is one of the most used plants, medically but also as an ingredient. Historically, garlic is originated from central Asia for over 6000 years ago [1]. Garlic is still used in folk medicine all over the world to treat a variety of diseases [2]. It has been extensively used throughout the history for its prophylactic and therapeutic effects. Its immunomodulatory and antitumor effects have also been demonstrated* in vitro *and* in vivo* [3]. The biological activity of garlic, which includes benefits in cardiovascular diseases, free radical scavenging, immune stimulating, anticancer, and anti-infectious properties, was shown in various studies [4–8].

## 2. Garlic Chemistry

More than 200 chemicals with diverse properties have been found in different garlic extracts [3]. Garlic possesses a higher concentration of sulfur compounds, which are responsible for the garlic flavor and health beneficial effects. It contains approximately 65% water, 28% carbohydrate, 2.3% organosulfur compounds, 2% proteins, 1.2% free amino acid, and 1.5% fibers.

The potential bioactive compounds present in garlic were divided into several groups [9]. The garlic chemical composition varies depending on whether it is intact or processed garlic. Garlic bioactive compounds could be divided into two major categories. The first category is nonvolatile sulfur-containing precursors in intact garlic. It carries about thirty-three sulfur compounds, several enzymes, and seventeen amino acids [10], in addition to steroidal glycosides and lectins [11, 12]. The major nonvolatile sulfur-containing precursor's compounds of this category are *γ*-glutamyl-*S*-allyl-L-cysteines and *S*-allyl-L-cysteine sulfoxides (alliin). Alliin is the precursor of allicin [13], methiin, (+)-*S*-(*trans*-1-propenyl)-L-cysteine sulfoxide, and cycloalliin [14]. The second category regroups organosulfur compounds, which are generated during the process of garlic product preparation. One of the most biologically active compounds, allicin (diallyl thiosulfinate or diallyl disulfide), is not found in garlic until it is “damaged”, crushed, cut, chewed, dehydrated, pulverized, or exposed to water which activates the enzyme alliinase that metabolizes alliin to allicin [15]. Allicin instantly decomposes to other compounds, such as diallyl sulfide (DAS), diallyl disulfide (DADS), dithiins, and ajoene. At the same time, *γ*-glutamyl cysteine is converted to *S*-allyl-cysteines (SAC), via a pathway other than the alliin–allicin's [9].

This category is divided into 3 groups depending on the chemical nature of the bioactive compounds: (1) thiosulfinates (e.g., allicin) result from the conversion of sulfoxides through an enzymatic reaction of sulfur-substituted cysteine sulfoxides when raw garlic is processed. Thus, no thiosulfinates are found in intact garlic. For instance, allicin is thought to be a transient compound that decomposes rapidly into other sulfur-containing compounds and is not found to be an active compound of garlic. *γ*-Glutamyl-*S*-allyl-L-cysteines are converted into SAC through an enzymatic transformation with *γ*-glutamyltranspeptidase when garlic is extracted with an aqueous solution. SAC is known for its biological activity [16, 17].

(2) Organosulfur volatiles: processed garlic contains a larger variety of organosulfur volatiles than the intact garlic. DAS, DADS, diallyl trisulfide, methylallyl disulfide, methylallyl trisulfide, 2-vinyl-4H-1, 3-dithiin, 3-vinyl-4H-1, 2-dithiin, and (E,Z)-ajoenes, which are typical volatiles, have been identified in crushed garlic and garlic essential oil. Furthermore, more than 20 sulfides have been identified in steam-distilled garlic oil and oil-soluble extract of garlic, and many of them, especially sulfides having an allyl group, are responsible for the specific smell and taste of garlic. The major sulfides in garlic oil include DAS (57%), allylmethyl (37%), and dimethyl (6%) mono- to hexasulfides, together with a small amount of allyl 1-propenyl and methyl 1-propenyl di-, tri-, and tetrasulfides [17]. Diallyl trisulfide is the most abundant in fresh garlic oil. The component of these sulfides varies according to extraction temperature and duration [17, 18].

(3) Water soluble organosulfur compounds: alcoholic and aqueous garlic extracts contain primarily S-allyl-L-cysteines derived from *γ*-glutamyl-S-allyl-L-cysteines. S-Allyl-L-cysteine and trans-S-1-propenyl-L-cysteine, together with a small amount of S-methyl-L-cysteine, are found in aged garlic extract [17].

Other bioactive compounds found in garlic are, for example, lectins, prostaglandins, pectin, adenosine, fructan, vitamins B1, B2, B6, C, and E, biotin, nicotinic acid, glycolipids, phospholipids, fatty acids, and essential amino acids in addition to sapogenins and steroid saponins to which some important pharmacological activities have been attributed such as antifungal, antitumor, antithrombotic, and hypocholesterolemic [19].

Various studies have shown that aged garlic extract exhibits higher biological properties compared to fresh garlic, garlic powder, and related formulations [20]. Garlic oil contains, mainly, diallyl disulfide, diallyl trisulfide, allyl propyl disulfide, disulfide, and smaller amounts of diallyl polysulfide [21]. The large varieties of effects reported with different garlic preparations might be due to these numerous compounds contained in different doses, depending on the preparation method. As a consequence, it is a challenge to separate and identify compounds with a given specific potential therapeutic activity [22].

Indeed, S-allyl-L-cysteine sulfoxide (alliin) and *γ*-glutamyl cysteine derivatives are the main compounds of fresh bulbs, while the sulfide family components take the first place as main component of steam-distilled oils. Powder from crushed and dried garlic contains alliin and diallyl disulfide (DADS). Macerates (ground garlic) are enriched extractions with sulfide family compounds, dithiins, and (E–Z)-ajoene compounds. Aged preparations (soaked, sliced, and aged garlic extract in ethanol solution) contain S-allyl-L-cysteine (SAC) and S-allyl mercaptocysteine (SAMC) [23]. Moreover, the proportion of these compounds is poorly controlled by the extraction approach. Therefore, the main challenge is reproducibility and subsequent validation of the effects observed by independent laboratories [24].

## 3. Garlic Preparation Biological Activities

### 3.1. Antioxidant Activity

Garlic extracts were found to have higher antioxidant potential than fresh garlic and other garlic preparations, which might be due to the presence of water soluble organosulfur compounds, such as SAC and SAMC, known for a great antioxidant potential [25]. The presence of flavonoids, saponins, some essential micronutrients, and macronutrients in garlic preparations may act synergistically in exerting their antioxidant potential by scavenging Reactive Oxygen Species (ROS) [26]. This antioxidant propriety is involved in the protection from oxidation of Deoxyribonucleic Acid (DNA), lipid, and protein by ROS which plays an important role in various diseases, including aging, cancer, inflammation, and neurodegeneration [1]. Phytochemicals in garlic extracts exercise their protection by enhancing the cellular antioxidant enzymes such as superoxide dismutase, catalase, and glutathione peroxidase and by increasing glutathione in the cells [27], an important defense mechanism in living cells [28]. Some of the potential benefits of garlic extract antioxidant propriety include anti-inflammatory effect by inhibiting oxidative stress-induced activation of nuclear factor-*κ*B (NF-*κ*B), which is the major transcription factor involved in the expression of proinflammatory enzymes such as Nitric Oxide Synthase (NOS) and cyclooxygenase-II [29]. The antioxidant potency of various garlic compounds differs based on its chemical structure and standardization procedures followed [17, 30]. The organosulfur compounds present in garlic are the potent antioxidants which also help to stimulate antioxidant enzymes in the liver [31]. For instance, heated garlic extract pretreatment of rats, which have been challenged with Cadmium (Cd), significantly increased antioxidative stress enzymes, liver SOD, and catalase [32]. Furthermore, garlic protects cells from oxidative injuries via induction of glutamate-cysteine ligase (GCL) through the increase of glutathione (GSH) content [32]. It has also been shown that ajoene (extracted from garlic) activates nuclear factor erythroid-2-related factor 2 (Nrf2), which regulates the expression of GCL and other cysteine-metabolizing enzymes genes. This was revealed by increased phosphorylation and nuclear accumulation of Nrf2, decreased interaction with Kelch-like ECH-associated protein-1, and decreased Nrf2 ubiquitination [33]. As a consequence, treatment of ajoene increased the antioxidant activity through augmentation of GCL mRNA and protein synthesis [33].

The antioxidant property plays an important role in the anticancer effect as well, since it inhibits free radical and mutation-mediated DNA damage [28]. Moreover, aged garlic extract was found to have radioprotective effects [34], protecting against ionizing radiation and UV light-induced DNA damage [35].

### 3.2. Modulation of Immune Cell Response

Garlic is considered as a capable candidate for maintaining the homeostasis of the immune system. Thus different studies have demonstrated interesting beneficial effects of garlic on the immunity and immune cells. For instance, it has been found that garlic consumption boosted mice immune cells and enhanced cells account in bone marrow [36]. It has been found that garlic protein fraction has a stimulatory effect on lymphocyte, Natural Killer (NK) cells, and macrophages cytotoxicity [37, 38]. Another group has also studied the effect of aqueous garlic extract and its protein fraction on macrophage and T cell functions in mice. They have observed a significant dose-dependent augmentation of oxidative burst of macrophages with both the garlic extract and the protein fraction and an enhancement of T cell proliferation in splenocytes stimulated with phytohemagglutinin (PHA), using the protein fraction [39]. These results go along with those found by Colic et al., in which they showed that higher doses of garlic extracts had an inhibitory effect while lower doses significantly enhanced lymphocyte proliferation upon stimulation with Concanavalin A (Con A) [40]. But these extracts used alone were not mitogenic for lymphocytes using rat splenocytes and thymocytes cultures. It has been shown also that three proteins purified from raw garlic extract exhibited a mitogenic activity towards human peripheral blood lymphocytes, murine splenocytes, and thymocytes [41]. Additionally, a protein fraction of fresh garlic was able to activate peripheral blood T lymphocyte and enlarged CD8+ T cell proliferation in treated animals, causing an increase in hypersensitivity delayed-type responses, promoting an efficient cellular response [42]. In the same direction, *γδ*T cell population, the only type of T cell that recognizes and responds to pathogen-associated molecular patterns (PAMP), increased its proliferation after aged garlic extract supplementation in healthy humans [43]. However, data from our group showed that garlic extract did not seem to have any effect neither on spontaneous nor PHA-induced proliferation of human CD4+ and CD8+ T lymphocytes [44]. As for other garlic compounds, like sulfur-containing components, these also showed a modulatory effect on T cell proliferation. Indeed, Feng et al. demonstrated that diallyl trisulfide (DATS) had a dual role on T lymphocyte proliferation in mice [45]. At higher concentration (50 *μ*g/ml), DATS inhibited T cell proliferation triggered by Con A; however, at lower concentrations (3–12.5 *μ*g/ml), it augmented the proliferative response of T cells to Con A. This dual effect was also confirmed with another garlic extract [40]. Compounds, such as ajoenes and alliin, isolated from garlic ethanol extracts selectively inhibited the proliferative response of human peripheral blood cells to lectins [46]. Surprisingly, Zamani et al. showed that garlic possesses the ability to increase lymphocyte proliferation* in vivo* in the absence of a mitogen [47]. This group showed that garlic aqueous solution induced an enhancement in lymphocyte proliferation in the spleen and thymus of rats from the garlic fed relative to the control group. This stimulatory effect was stronger in thymocytes compared with splenocytes, and nuclei sizes were also smaller in thymocytes due to increased cell proliferation [47]. Data are summarized in Table 1 about immune cell response.

As for cytokine expression and production, Allicin, as other organosulfur compounds, was found to inhibit Th1 proinflammatory cytokines [48]. Other* in vivo* studies showed that garlic oil gavage in rats had a double effect on the T helper (Th1/Th2) balance. At low doses, T cell response was enhanced towards the Th1 type; while at high doses, it triggered the Th2 type [49], this dual effects might be due to the presence of different receptors with distinct affinities, although there is no evidence, at the best of our knowledge, in the literature for this specific example. In a similar work by Zamani et al., oral garlic treatment seemed to favor a Th2 response by inducing an increased IL-4 production in spleen lymphocytes of the treated rats [50]. Another study in mice treated with aged garlic extract showed a capacity of alteration in normal cytokine production towards a Th1 response and a reduced number of T regulatory cells (Treg), a protective pattern, which is beneficial to the antitumor immunity [51]. These data go along with those found by Ota et al. while testing the effect of garlic extracts on Peyer's patches of mice intestine which revealed a higher production of IFN-*γ* and IL-4 on the contrary to IL-2 production [52]. However, in another study, using peripheral blood monocytes, aged garlic extract upregulated IL-10 that acts as negative feedback in the proinflammatory response signaling by inhibiting the production of the proinflammatory cytokines TNF-*α* and IL-6 [48]. In the same direction, the aged extract decreased IL-12 production that could cause a downregulation of other proinflammatory cytokines (interferon gamma “IFN-*γ*” and interleukin-2 “IL-2”) produced by T cells [48, 53]. Keiss et al. have shown that garlic powder extracts modulated lipopolysaccharide-induced cytokine levels in human whole blood, such as reducing proinflammatory cytokine like IL-1*β* and TNF-*α*, whereas the expression of the anti-inflammatory cytokine IL-10 was unchanged [54]. These data are consistent with ours, where we showed that human Peripheral Blood Mononuclear Cells (PBMCs) treated with garlic extract did not affect IL-4 expression while inhibiting significantly the proinflammatory cytokine IL-17 [44]. These immunomodulatory properties of garlic could be useful in clinical applications, since it enhances innate and specific cell immunity and also improves host resistance. It was also reported that allicin modulates T cells and adhesion molecules and exerts an inhibitory effect on NF-*κ*B activation and hence prevents liver damage [55, 56]. Moreover, other compounds, such as allicin, exert negative effects on human T cell migration through fibronectin by downregulating actin reorganization [57]. Furthermore, a protein fraction, isolated from aged garlic extract, enhances the cytotoxic activity of human peripheral blood lymphocytes in synergy with IL-2 and independently from INF-*γ* or TNF-*α* [38]. Other garlic derived compounds, such as caffeic acid, SAC, and DATS, can inhibit the transcription factor NF-*κ*B, which results in the inhibition of the transcription of several cytokine genes involved in proinflammatory responses, such as TNF-*α*, IL-1*β*, IL-6, MCP-1, and IL-12(p70) [58–60]. Targeting cytokine expression by natural compounds, in order to modulate the global immune response, may represent an interesting therapeutic approach [61]. Data about cytokine expression and production are summarized in Table 2.

### 3.3. Effect of Garlic on Inflammatory Diseases

Garlic therapeutic potential has been studied in several pathologies. In particular, in inflammatory diseases, its effect on immune system components is associated with the proinflammatory state, related to the induction of inflammatory mediators, the oxidative stress, and the activation of different immune cells. For instance, garlic extract was found to decrease significantly allergic-airway inflammation in a mouse allergic-airway inflammation model [62]. As for inflammatory bowel disease, which is characterized by a predominantly Th1-mediated response, it has been reported that garlic or its compounds modulated leukocyte proliferation and cytokine production. Th1 cell inflammatory cytokine production is reduced significantly, while IL-10 production is upregulated; in addition to IFN-*γ* and IL-2, TNF-*α* is also significantly inhibited in the presence of garlic extract and/or its compounds, which revealed a potential therapeutic use in inflammatory conditions such as inflammatory bowel disease [15, 48]. Therefore, garlic can be considered as an excellent preventive and protective agent against gastric inflammatory pathologies.

The anti-inflammatory effects of garlic extracts result from its dual direct effect on IL-10 and IL-12 in inflammatory bowel disease. And the indirect effect on IFN-*γ* production in T and NK cells occurs [24, 48]. Allicin, an important compound of garlic, inhibited the TNF-*α* secretion, supporting the anti-inflammatory effect of allicin on intestinal epithelial cells [63]. SAC exhibited a dose-dependent inhibition of NF*κ*B activation induced by both TNF-*α* and H_2_O_2_ in human T lymphocytes, Jurkat cells [64].

Thiacremonone, a sulfur compound isolated from garlic, was found to inhibit iNOS expression and NO^∙^ production through blocking NF*κ*B activity* in vitro* and to ameliorate inflammatory responses and arthritic reactions in acute and chronic edema and arthritic animal models [65, 66]. In an attempt to explain the molecular mechanisms of NF*κ*B inhibition, investigators demonstrated that sulfur compounds react with cysteine residues of target molecules in the intracellular signal transduction proteins through cysteine-cysteine interaction, thus inhibiting the inflammatory responses and development of arthritic rheumatism [67–69]. These data suggest that thiacremonone may be potentially beneficial for the prevention of inflammatory diseases such as arthritic rheumatism [65].

Liu et al. found an hypoglycemic effect of garlic oil, associated with a lowered level of NO in skeletal muscle [70], which is consistent with the finding of another study showing that, in diet-induced metabolic syndromes in rats, treatment with raw garlic homogenate efficiently improves insulin sensitivity and normalizes serum levels of nitric oxide [71]. These hypoglycemic effects of garlic products might be explained partially by inhibition of proinflammatory mediators. In fact, inflammation could trigger insulin resistance, and the identified inflammatory factors involved in this process include molecules such as TNF-*α*, IL-1*β*, and NO [70, 72, 73].

Several studies* in vitro *have confirmed the cardioprotective effect of garlic on primary cultured cardiac myocytes, fibroblasts, and endothelial cells, by reducing the production of ROS and blocking ROS-dependent activation of extracellular signal-regulated kinase [74] 1/2, JNK1/2, AKT, NF*κ*B, and SMADS signaling [31, 60, 75].

Atherosclerosis is known as a complex pathology characterized by an excessive inflammatory and proliferative response to damage in the vascular endothelium and involving several cell types, particularly smooth muscle cells, monocyte-derived macrophages, T lymphocytes, and platelets [76, 77].

Garlic compound, 1, 2-vinyldithiin, was found to reduce the secretion of IL-6, in human preadipocytes treated with macrophage factors, which is associated with low-grade chronic inflammation and metabolic complications of obesity [78]. This inflammation is characterized by abnormal cytokine production that increased in the acute phase by infiltrated macrophage, mast-cell, and NK cell in the adipose tissue [79–81]. In the same direction, alliin was found to prevent the increase of gene expression and proteins related to the proinflammatory state induced by LPS in 3T3-L1 adipocytes. This effect was through the toll-like receptor-4 (TLR-4) pathway and possibly by regulating ERK1/2 activity [82]. Another study has demonstrated that garlic extract suppressed LPS-induced TLR4 dimerization, suggesting this inhibition to be one of the mechanisms for the garlic anti-inflammatory activity [83]. These data showed that garlic can modulate inflammatory responses through the suppression of TLR activation leading to the inhibition of NF*κ*B and COX-2 activation and iNOS expression. These results provide new insight into understanding the mechanism by which garlic extract exerts anti-inflammatory effects.

### 3.4. Role of Garlic in Antitumor Immunity

Several studies have indicated that intake of garlic reduces both carcinoma and sarcoma risk in different tissues and body organs, such as bladder, colon, prostate, lung, oesophagus, stomach, skin, brain, and liver [84]. Other studies have demonstrated the role of protein fractions from garlic bulbs in tumor growth, since a significant decrease in the size of mouse mammary tumor [85] and complete suppression of growth of Human Erythroleukemia cell line (HEL), in a dose-dependent manner, were found [86]. The mechanisms of these effects are not fully understood. Garlic antitumor potential has also been shown both* in vitro *and* in vivo*. For instance, it was found that garlic increases frequency and function of NK cells [37, 38, 87], a cell type known to play major roles in antitumor immunity. Garlic also enhances the frequency and proliferation of lymphocytes [88, 89] and normalizes CD4+/CD8+ T cell ratio (increased T CD4+ and decreased TCD8+) [90]. Other reports showed that garlic exhibits antiproliferative and antiangiogenesis effects on tumor cells [91, 92]. Moreover, it is found that garlic extracts inhibited completely growth of implanted tumor cells which were directly preexposed to garlic extracts [93]. Dietary administration led to an enhanced NK activity in peripheral bloods of animal models. In 1967, Fujiwara and Natata have tried to induce tumor immunity using tumor cells treated with extract of garlic [94]. Slight delay in tumor appearance and animal death through administration of garlic extract has also been reported by Aboul-Enein in 1986 [95]. It has also been shown by Hu et al. in 2002 using an* in vitro *assessment that direct exposure of tumor cells to aged garlic extract results in a suppression of tumor cell growth and migration [93].

Fallah-Rostami et al. findings showed that the administration of aged garlic extract induced effective immune responses against fibrosarcoma tumor in BALB/c mice and led to significant inhibition of tumor growth and enhanced mice survival time [3]. They found significant increase in splenocytes IFN-*γ* production from aged garlic extract treated mice. IFN-*γ* secretion by CD4+ Th1 cells, CD8+ cells, *γδ*T cells, and activated NK cells plays an important role in activating lymphocytes to enhance tumor immunosurveillance. Other studies showed that proteins isolated from garlic modulate NK cell line activity in the mesenteric lymph node of mouse [43]. Therefore, garlic acts as a proliferation inducer for this cell population [85], while aged garlic extract modulates the number and the activity of NK cells in patients with various advanced cancers [37] and also increases NK activities against different cancer cell lines [96]. However, results differ from one study to another. In Ishikawa et al. report, it was shown that administering aged garlic extract to patients with advanced cancer of the digestive system improved NK cell activity but caused no improvement in T lymphocyte proliferation [37]. However, in healthy subjects, aged garlic extract increases the NK cell population [24].

The results published by Zamani et al. are inconsistent with the well-recognized antiproliferative effect of garlic in several tumor cells [37, 47, 87, 97, 98]. Since the authors showed that garlic may enhance rat splenocytes and thymocytes proliferation. This may be due to the metabolic differences between tumor and normal cells [99].

As for cytokine production, a decrease in TNF-*α* levels and an increase of cytotoxic damage markers were observed in Ehrlich Ascites Carcinoma (EAC) cells treated with tamoxifen and supplemented with allicin [100]. Other studies have demonstrated the role of protein fractions from garlic bulbs in tumor growth and intratumor-infiltrated T lymphocytes in mice transplanted with mammary tumor cells [42]. Other groups have shown that a fraction of aged garlic extract, combined with IL-2 administration, could be employed in tumor immunotherapy, because this combination increases the cytotoxicity of T cell lineage [38]. It has also been proposed that garlic organosulfur compounds could inhibit the sulfhydryl group in proteins hydrophobic parts and cysteine residues in hormone binding site within oestrogen receptors (e.g., allyl sulfides) [86]. Interestingly, Larypoor et al. suggested that aged garlic extract could be used as herbal medicine with few limited side effects as compared to chemotherapy, which is conventionally used in treating cancers [51], since aged garlic extract was found to be able to alter cytokine production in normal mice towards a Th1 protective pattern with potential antitumor immunity properties. Another study showed that there was a significant increase in apoptosis of acute lymphoblastic leukemia cells with no alteration of T cell activation as determined by CD25/CD69 upregulation and IFN-*γ*, IL-2, and TNF-*α* production [66, 101]. In contrast, the presence of chemotherapeutic agents resulted in nonselective increases in both lymphocyte and acute lymphoblastic leukemia apoptosis and a decrease in T cell proliferation and cytokine production [101].

Protective effects of garlic against cancer might be due to its ability to block the activation and formation of cancer causing substances and enhance DNA repair, attenuation of ROS formation, reduction or inhibition of cell proliferation, or induction of cell death and instruction of efficient antitumor immunity [102–105]. The anticancer effects of garlic are being extensively documented and most of these effects have been attributed to the organosulfur compounds such as diallyl sulfide, diallyl disulfide, diallyl trisulfide, S-allylcysteine, or S-allylmercaptocysteine [106]. Recent findings have shown that the antitumor effect of allyl sulfur compounds may be related to their anti-inflammatory as well as immune-stimulatory properties [107].

## 4. Concluding Remarks

Garlic is one of the most used flavoring plants for cooking. Garlic has a long history being used in traditional medicine with protective and curative purposes. Garlic or its different bioactive molecules and formulations have been extensively probed in* in vitro *and* in vivo *studies to examine anti-inflammatory and immunomodulatory properties. One of the main mechanisms observed is through modulation of cytokine profiles and, on the other hand, direct instruction and stimulation of immune cells. It is suggested that the garlic beneficial effects are attributed, in particular, to sulfur-containing compounds, some polyphenols, and flavonoids. The synergistic effect of the different compounds present in garlic preparations might be responsible for the biologic activities revealed in different pathological situations. However, the identification of the potential compound(s), which could eventually mediate efficient antitumor immunity, would be of major interest.

## Figures and Tables

**Table 1:** Principal biological effects of garlic or its compounds on immune cell activation and proliferation.

Garlic products	The biological effect on immune cell proliferation	Authors
Garlic consumption	Enhancement of cells account in bone marrow	[36]
	Increasing lymphocyte proliferation *in vivo* in the absence of a mitogen Enhancement in lymphocyte proliferation in the spleen and thymus of rats from the garlic fed relative to the control group	[47]

Protein fraction	Stimulatory effect on lymphocyte and NK and macrophages cytotoxicity	[37, 38]

Extract and protein fraction	A dose-dependent augmentation of oxidative burst of macrophages An enhancement of T cell proliferation in splenocytes stimulated with PHA	[39]

Proteins from raw garlic extract	Mitogenic activity towards human peripheral blood lymphocytes, murine splenocytes, and thymocytes	[41]
Protein fraction of fresh garlic	Activating peripheral blood T lymphocyte and enlarged CD8+ T cell proliferation	[42]

Aged extract consumption	Increasing its proliferation of *γδ*T cell population in healthy humans	[43]

Diallyl trisulfide (DATS)	Dual effect (inhibition and augmentation) on T lymphocyte proliferation in mice	[40, 45]

Ajoenes and alliin	Selective inhibition of proliferative response of human peripheral blood cells to lectins	[46]

**Table 2:** Garlic effects on cytokine expression and production.

Garlic products	The biological effect on cytokine expression and production	Authors
Allicin	Inhibiting Th1 proinflammatory cytokines	[48]
	An inhibitory effect on NF-*κ*B activation	[55, 56]
	A negative effect on human T cell migration	[57]

Garlic oil gavage	In rats: At low doses, enhancement of T cell response towards the Th1 type At high doses, it triggered the Th2 type	[49]

Oral garlic consumption	Favoring a Th2 response via inducing an increased IL-4 production in spleen lymphocytes of the treated rats	[50]

Aged garlic extract	Alteration in normal cytokine production to a Th1 response in mice IL-10 upregulation in peripheral blood monocytes	[51]
	Decreasing IL-12 production	[48, 53]

Garlic extracts	Increasing production of IFN-*γ* and IL-4 and reducing IL-2 production in Peyer's patches of mice intestine	[52]
	Inhibition of IL-17 expression in treated human PBMCs	[44]

Garlic powder extracts	Reducing proinflammatory cytokine like IL-1*β* and TNF-*α*, without changing IL-10 level human whole blood	[54]

## References

[1] Rasul S. H. A., Sadiq B. M., Nauman K. (2015). Garlic (Allium sativum): diet based therapy of 21st centurya review. *Asian Pacific Journal of Tropical Disease*.

[2] Ali M., Thomson M., Afzal M. (2000). Garlic and onions: their effect on eicosanoid metabolism and its clinical relevance. *Prostaglandins, Leukotrienes and Essential Fatty Acids*.

[3] Fallah-Rostami F., Tabari M. A., Esfandiari B., Aghajanzadeh H., Behzadi M. Y. (2013). Immunomodulatory activity of aged garlic extract against implanted fibrosarcoma tumor in mice. *North American Journal of Medicine & Science*.

[4] Singh B. B., Vinjamury S. P., Der-Martirosian C. (2007). Ayurvedic and collateral herbal treatments for hyperlipidemia: A systematic review of randomized controlled trials and quasi-experimental designs. *Alternative Therapies in Health and Medicine*.

[5] Borek C. (2006). Garlic reduces dementia and heart-disease risk. *Journal of Nutrition*.

[6] Khanum F., Anilakumar K. R., Viswanathan K. R. (2004). Anticarcinogenic properties of garlic: a review. *Critical Reviews in Food Science and Nutrition*.

[7] Harris J. C., Cottrell S. L., Plummer S., Lloyd D. (2001). Antimicrobial properties of Allium sativum (garlic). *Applied Microbiology and Biotechnology*.

[8] Colic M., Vucevic D., Kilibarda V., Radicevic N., Savic M. (2000). Garlic extracts stimulate proliferation of rat lymphocytes in vitro by increasing il-2 and il-4 production. *Immunopharmacology and Immunotoxicology*.

[9] Rana S. V., V S., Pal R., Vaiphei K., Sharma S. K., Ola R. P. (2011). Garlic in health and disease. *Nutrition Research Reviews*.

[10] Fenwick G. R., Hanley A. (1985). The genus Allium. Part 2. *Critical Reviews in Food Science and Nutrition*.

[11] Kaku H., Goldstein I. J., Van Damme E. J. M., Peumans W. J. (1992). New mannose-specific lectins from garlic (Allium sativum) and ramsons (Allium ursinum) bulbs. *Carbohydrate Research*.

[12] Matsuura H., Ushiroguchi T., Itakur Y., Hayashi N., Fuwa T. (1988). A Furostanol Glycoside from Garlic, Bulbs of Allium sativum L.. *Chemical & Pharmaceutical Bulletin*.

[13] Stoll A., Seebeck E. (1948). Allium compounds. I. Alliin the true mother compound of garlic oil. *Helv Chim Acta*.

[14] Fujiwara M., Yishimura M., Tsuno S., Murakami F. (1958). “allithiamine”, a newly found derivative of vitamin B1: IV. on the alliin homologues in the vegetables. *The Journal of Biochemistry*.

[15] Londhe V. P., Gavasane A. T., Nipate S. S., Bandawane D. D., Chaudhari P. D. (2011). Role of garlic (*Allium sativum*) in various diseases: an overview. *Journal of Pharmaceutical Research and Opinion*.

[16] Matsuura H. (1997). Phytochemistry of garlic horticultural and processing procedures. *Lachance PA,editor. Neutraceuticals: designer foods III. garlic, soy and licorice*.

[17] Amagase H. (2006). Clarifying the real bioactive constituents of garlic. *Journal of Nutrition*.

[18] Block E. (1992). The organosulfur chemistry of the genus Allium—implications for the organic chemistry of sulfur. *Angewandte Chemie International Edition*.

[19] Suleria H. A. R., Butt M. S., Anjum F. M., Sultan S., Khalid N. (2013). Aqueous garlic extract; natural remedy to improve haematological, renal and liver status. *Journal of Nutrition and Food Sciences*.

[20] Banerjee S., Mukherjee P., Maulik S. (2003). Garlic as an antioxidant: the good, the bad and the ugly. *Phytotherapy Research*.

[21] Pranoto Y., Salokhe V. M., Rakshit S. K. (2005). Physical and antibacterial properties of alginate-based edible film incorporated with garlic oil. *Food Research International*.

[22] Sivam G. P. (2001). Protection against Helicobacter pylori and Other Bacterial Infections by Garlic. *Journal of Nutrition*.

[23] Trio P. Z., You S., He X., He J., Sakao K., Hou D.-X. (2014). Chemopreventive functions and molecular mechanisms of garlic organosulfur compounds. *Food & Function*.

[24] Arreola R., Quintero-Fabian S., Lopez-Roa R. I. (2015). Immunomodulation and anti-inflammatory effects of garlic compounds. *Journal of Immunology Research*.

[25] Ichikawa M., Yoshida J., Ide N., Sasaoka T., Yamaguchi H., Ono K. (2006). Tetrahydro-*β*-carboline derivatives in aged garlic extract show antioxidant properties. *Journal of Nutrition*.

[26] Borek C. (2001). Antioxidant Health Effects of Aged Garlic Extract. *Journal of Nutrition*.

[27] Liu J. Z., Lin X. Y., Milner J. A. (1992). Dietary garlic powder increases glutathione content and glutathione S-transferase activity in rat liver and mammary tissues. *The FASEB Journal*.

[28] Borek C. (1997). Antioxidants and cancer. *Science & Medicine*.

[29] Santhosha S. G., Jamuna P., Prabhavathi S. N. (2013). Bioactive components of garlic and their physiological role in health maintenance: A review. *Food Bioscience*.

[30] Chung L. Y. (2006). The antioxidant properties of garlic compounds: Alyl cysteine, alliin, allicin, and allyl disulfide. *Journal of Medicinal Food*.

[31] Ide N., Lau B. H. S. (2001). Garlic compounds minimize intracellular oxidative stress and inhibit nuclear factor-*κ*B activation. *Journal of Nutrition*.

[32] Park J.-H., Park Y. K., Park E. (2009). Antioxidative and antigenotoxic effects of garlic (Allium sativum L.) prepared by different processing methods. *Plant Foods for Human Nutrition*.

[33] Kay H. Y., Yang J. W., Kim T. H. (2010). Ajoene, a stable garlic by-product, has an antioxidant effect through Nrf2-mediated glutamate-cysteine ligase induction in HepG2 cells and primary hepatocytes. *Journal of Nutrition*.

[34] Lau B. H. S. (1989). Detoxifying, radioprotective and phagocyte enhancing effects of garlic. *International Clinical Nutrition Review*.

[35] Corzomartinez M., Corzo N., Villamiel M. (2007). Biological properties of onions and garlic. *Trends in Food Science & Technology*.

[36] Kuttan G. (2000). Immunomodulatory effect of some naturally occuring sulphur-containing compounds. *Journal of Ethnopharmacology*.

[37] Ishikawa H., Saeki T., Otani T. (2006). Aged garlic extract prevents a decline of NK cell number and activity in patients with advanced cancer. *The Journal of Nutrition*.

[38] Morioka N., Szel L. L., Morton D. L., Irie R. F. (1993). A protein fraction from aged garlic extract enhances cytotoxicity and proliferation of human lymphocytes mediated by interleukin-2 and concanavalin A. *Cancer Immunology, Immunotherapy*.

[39] Lau B. H., Yamasaki T., Gridley D. S. (1991). Garlic compounds modulate macrophage and T-lymphocyte functions. *Molecular Biotherapy*.

[40] Colic M., Vucevic D., Kilibarda V., Radicevic N., Savic M. (2002). Modulatory effects of garlic extracts on proliferation of T-lymphocytes in vitro stimulated with concanavalin A. *Phytomedicine*.

[41] Clement F., Pramod S. N., Venkatesh Y. P. (2010). Identity of the immunomodulatory proteins from garlic (Allium sativum) with the major garlic lectins or agglutinins. *International Immunopharmacology*.

[42] Ebrahimi M., Hassan Z. M., Mostafaie A., Mehrjardi N. Z., Ghazanfari T. (2013). Purified protein fraction of garlic extract modulates cellular immune response against breast transplanted tumors in BALB/c mice model. *Cell*.

[43] Nantz M. P., Rowe C. A., Muller C. E., Creasy R. A., Stanilka J. M., Percival S. S. (2012). Supplementation with aged garlic extract improves both NK and *γδ*-T cell function and reduces the severity of cold and flu symptoms: a randomized, double-blind, placebo-controlled nutrition intervention. *Clinical Nutrition*.

[44] Moutia M., Seghrouchni F., Abouelazz O. (2016). Allium sativum L. regulates in vitro IL-17 gene expression in human peripheral blood mononuclear cells. *BMC Complementary and Alternative Medicine*.

[45] Feng Z. H., Zhang G. M., Hao T. L., Zhou B., Zhang H., Jiang Z. Y. (1994). Effect of diallyl trisulfde on the activation of T cell and macrophage-mediated cytotoxicity. *Journal of Tongji Medical University*.

[46] Romano E. L., Montaño R. F., Brito B. (1997). Effects of Ajoene on lymphocyte and macrophage membrane-dependent functions. *Immunopharmacology and Immunotoxicology*.

[47] Zamani A., Hoseinipanah M., Madadi H., Arjipour M. (2011). Effect of garlic consumption on the argyrophilic nucleolar organiser regions (AgNORs) in splenocytes and thymocytes of rats. *Food and Agricultural Immunology*.

[48] Hodge G., Hodge S., Han P. (2002). *Allium sativum* (garlic) suppresses leukocyte inflammatory cytokine production in vitro: potential therapeutic use in the treatment of inflammatory bowel disease. *Cytometry*.

[49] Liu C. T., Su H. M., Lii C. K., Sheen L.- Y. (2009). Effect of supplementation with garlic oil on activity of Th1 and Th2 lymphocytes from rats. *Planta Medica*.

[50] Zamani A., Vahidinia A., Ghannad M. S. (2009). The effect of garlic consumption on TH1/TH2 cytokines in phytohemagglutinin (PHA) activated rat spleen lymphocytes. *Phytotherapy Research*.

[51] Larypoor M., Bayat M., Hassan Z. M., Sepahy A. A., Amanlou M. (2013). Evaluation of the number of CD4+ CD25+ FoxP3 + Treg cells in normal mice exposed to AFB1 and treated with aged garlic extract. *Cell*.

[52] Ota N., Takano F., Muroga S. (2012). Garlic extract and its selected organosulphur constituents promote ileal immune responses ex vivo. *Journal of Functional Foods*.

[53] Gazzinelli R. T., Oswald I. P., James S. L., Sher A. (1992). IL-10 inhibits parasite killing and nitrogen oxide production by IFN-*γ*-activated macrophages. *The Journal of Immunology*.

[54] Keiss H.-P., Dirsch V. M., Hartung T. (2003). Garlic (*Allium sativum* L.) modulates cytokine expression in lipopolysaccharide-activated human blood thereby inhibiting NF-*κ*B activity. *Journal of Nutrition*.

[55] Dorhoi A., Dobrean V., Zǎhan M., Virag P. (2006). Modulatory effects of several herbal extracts on avian peripheral blood cell immune responses. *Phytotherapy Research*.

[56] Bruck R., Aeed H., Brazovsky E., Noor T., Hershkoviz R. (2005). Allicin, the active component of garlic, prevents immune-mediated, concanavalin A-induced hepatic injury in mice. *Liver International*.

[57] Sela U., Ganor S., Hecht I. (2004). Allicin inhibits SDF-1alpha-induced T cell interactions with fibronectin and endothelial cells by down-regulating cytoskeleton rearrangement, Pyk-2 phosphorylation and VLA-4 expression. *The Journal of Immunology*.

[58] Ho C. Y., Weng C. J., Jhang J. J., Cheng Y. T., Huang S. M., Yen G. C. (2014). Diallyl sulfide as a potential dietary agent to reduce TNFalpha- and histamine-induced proinflammatory responses in A7r5 cells. *Molecular Nutrition & Food Research*.

[59] You S., Nakanishi E., Kuwata H. (2013). Inhibitory effects and molecular mechanisms of garlic organosulfur compounds on the production of inflammatory mediators. *Molecular Nutrition Food Research*.

[60] Kim S. R., Jung Y. R., An H. J. (2013). Anti-wrinkle and anti-inflammatory effects of active garlic components and the inhibition of MMPs via NF-*κ*B signaling. *PLoS ONE*.

[61] Spelman K., Burns J. J., Nichols D., Winters N., Ottersberg S., Tenborg M. (2006). Modulation of cytokine expression by traditional medicines: a review of herbal immunomodulators. *Alternative Medicine Review*.

[62] Zare A., Farzaneh P., Pourpak Z. (2008). Purified aged garlic extract modulates allergic airway inflammation in Balb/c mice. *Iranian Journal of Allergy, Asthma and Immunology*.

[63] Lang A., Lahav M., Sakhnini E. (2004). Allicin inhibits spontaneous and TNF-*κ* induced secretion of proinflammatory cytokines and chemokines from intestinal epithelial cells. *Clinical Nutrition*.

[64] Geng Z., Rong Y., Lau B. H. S. (1997). S-Allyl Cysteine Inhibits Activation of Nuclear Factor Kappa B in Human T Cells. *Free Radical Biology and Medicine*.

[65] Ban J. O., Oh J. H., Kim T. M. (2009). Anti-inflammatory and arthritic effects of thiacremonone, a novel sulfur compound isolated from garlic via inhibition of NF-kappaB. *Arthritis Research & Therapy*.

[66] Shreffler W. G., Visness C. M., Burger M. (2006). Standardization and performance evaluation of mononuclear cell cytokine secretion assays in a multicenter study. *BMC Immunology*.

[67] Kim K.-M., Chun S.-B., Koo M.-S. (2001). Differential regulation of NO availability from macrophages and endothelial cells by the garlic component S-allyl cysteine. *Free Radical Biology & Medicine*.

[68] Lee K. S., Kim S. R., Park H. S. (2007). A novel thiol compound, N-acetylcysteine amide, attenuates allergic airway disease by regulating activation of NF-*κ*B and hypoxia-inducible factor-1*α*. *Experimental & Molecular Medicine*.

[69] Humar M., Dohrmann H., Stein P. (2007). Thionamides Inhibit the Transcription Factor Nuclear Factor- B by Suppression of Rac1 and Inhibitor of B Kinase. *The Journal of Pharmacology and Experimental Therapeutics*.

[70] Liu C.-T., Hsu T.-W., Chen K.-M., Tan Y.-P., Lii C.-K., Sheen L.-Y. (2012). The antidiabetic effect of garlic oil is associated with ameliorated oxidative stress but not ameliorated level of pro-inflammatory cytokines in skeletal muscle of streptozotocin-induced diabetic rats. *Journal of Traditional and Complementary Medicine*.

[71] Padiya R., Khatua T. N., Bagul P. K., Kuncha M., Banerjee S. K. (2011). Garlic improves insulin sensitivity and associated metabolic syndromes in fructose fed rats. *Journal of Nutrition and Metabolism*.

[72] Jager J., Grémeaux T., Cormont M., Le Marchand-Brustel Y., Tanti J.-F. (2007). Interleukin-1*β*-induced insulin resistance in adipocytes through down-regulation of insulin receptor substrate-1 expression. *Endocrinology*.

[73] Tilg H., Moschen A. R. (2008). Inflammatory mechanisms in the regulation of insulin resistance. *Molecular Medicine*.

[74] Mithen R. F., Dekker M., Verkerk R., Rabot S., Johnson I. T. (2000). The nutritional signifcance, biosynthesis and bioavailability of glucosinolates in human foods. *Journal of the Science of Food and Agriculture*.

[75] Liu C., Cao F., Tang Q. Z. (2010). Allicin protects against cardiac hypertrophy and fibrosis via attenuating reactive oxygen species-dependent signaling pathways. *The Journal of Nutritional Biochemistry*.

[76] Morihara N., Ide N., Weiss N. (2011). Aged garlic extract inhibits homocysteine-induced scavenger receptor CD36 expression and oxidized low-density lipoprotein cholesterol uptake in human macrophages in vitro. *Journal of Ethnopharmacology*.

[77] Sitia S., Tomasoni L., Atzeni F. (2010). From endothelial dysfunction to atherosclerosis. *Autoimmunity Reviews*.

[78] Ried K., Frank O. R., Stocks N. P. (2010). Aged garlic extract lowers blood pressure in patients with treated but uncontrolled hypertension: a randomised controlled trial. *Maturitas*.

[79] Lumeng C. N., Deyoung S. M., Saltiel A. R. (2007). Macrophages block insulin action in adipocytes by altering expression of signaling and glucose transport proteins. *American Journal of Physiology-Endocrinology and Metabolism*.

[80] Liu J., Divoux A., Sun J. (2009). Genetic deficiency and pharmacological stabilization of mast cells reduce diet-induced obesity and diabetes in mice. *Nature Medicine*.

[81] Hotamisligil G. S. (2006). Inflammation and metabolic disorders. *Nature*.

[82] Quintero-Fabián S., Ortuño-Sahagún D., Vázquez-Carrera M., López-Roa R. I. (2013). Alliin, a garlic (Allium sativum) compound, prevents LPS-induced inflammation in 3T3-L1 adipocytes. *Mediators of Inflammation*.

[83] Youn H. S., Lim H. J., Lee H. J. (2008). Garlic (Allium sativum) extract inhibits lipopolysaccharide-induced Toll-like receptor 4 dimerization. *Bioscience, Biotechnology, and Biochemistry*.

[84] Chauhan N. (2005). Multiplicity of garlic health effects and Alzheimers disease. *The Journal of Nutrition Health and Aging*.

[85] Hassan Z. M., Yaraee R., Zare N., Ghazanfari T., Nejad A. H. S., Nozari B. (2003). Immunomodulatory affect of R10 fraction of garlic extract on natural killer activity. *International Immunopharmacology*.

[86] Sigounas G., Hooker J., Anagnostou A., Steiner M. (1997). S-allylmercaptocysteine inhibits cell proliferation and reduces the viability of erythroleukemia, breast, and prostate cancer cell lines. *Nutrition and Cancer*.

[87] Lamm D. L., Riggs D. (2000). The potential application of Allium sativum (garlic) for the treatment of bladder cancer. *Urologic Clinics of North America*.

[88] Cheng F., McLaughlin P. J., Banks W. A., Zagon I. (2009). Passive diffusion of naltrexone into human and animal cells and upregulation of cell proliferation. *AJP Regulatory Integrative and Comparative Physiology*.

[89] Zagon S., McLaughlin P. J. (1990). Opioid antagonist (naltrexone) stimulation of cell proliferation in human and animal neuroblastoma and human fibrosarcoma cells in culture. *Neuroscience*.

[90] Scifo R., Cioni M., Nicolosi A. (1996). Opioid-immune interactions in autism: Behavioural and immunological assessment during a double-blind treatment with naltrexone. *Annali Dell’Istituto Superiore Di Sanità*.

[91] Golovchenko I., Yang C.-H., Goalstone M. L., Draznin B. (2003). Garlic extract methylallyl thiosulfinate blocks insulin potentiation of platelet-derived growth factor-stimulated migration of vascular smooth muscle cells. *Metabolism - Clinical and Experimental*.

[92] Matsuura N., Miyamae Y., Yamane K. (2006). Aged garlic extract inhibits angiogenesis and proliferation of colorectal carcinoma cells. *The Journal of Nutrition*.

[93] Hu X., Cao B. N., Hu G., He J., Yang D. Q., Wan Y. (2002). Attenuation of cell migration and induction of cell death by aged garlic extract in rat sarcoma cells. *International Journal of Molecular Medicine*.

[94] Fujiwara M., Natata T. (1967). Induction of tumour immunity with tumour cells treated with extract of garlic (allium sativum). *Nature*.

[95] Aboul‐Enein A. M. (1986). Inhibition of tumor growth with possible immunity by Egyptian garlic extracts. *Food / Nahrung*.

[96] Kyo E., Uda N., Suzuki A. (1998). Immunomodulation and antitumor activities of aged garlic extract. *Phytomedicine*.

[97] Pinto J. T., Rivlin R. S. (2001). Antiproliferative effects of allium derivatives from garlic. *The Journal of Nutrition*.

[98] Rivlin R. S. (2009). Can garlic reduce risk of cancer?. *American Journal of Clinical Nutrition*.

[99] Velasco-Velázquez M. A., Maldonado P. D., Barrera D., Torres V., Zentella-Dehesa A., Pedraza-Chaverrí J. (2006). Aged garlic extract induces proliferation and ameliorates gentamicin-induced toxicity in LLC-PK1 cells. *Phytotherapy Research*.

[100] Suddek G. M. (2014). Allicin enhances chemotherapeutic response and ameliorates tamoxifen-induced liver injury in experimental animals. *Pharmaceutical Biology*.

[101] Hodge G., Davis S., Rice M., Tapp H., Saxon B., Revesz T. (2008). Garlic compounds selectively kill childhood pre-B acute lymphoblastic leukemia cells in vitro without reducing T-cell function: Potential therapeutic use in the treatment of ALL. *Biologics : Targets & Therapy*.

[102] Lvova G. N., Zasukhina G. D. (2002). Modification of repair DNA synthesis in mutagen-treated human fibroblasts during adaptive response and the antimutagenic effect of garlic extract. *Genetika*.

[103] Arnault I., Auger J. (2006). Seleno-compounds in garlic and onion. *Journal of Chromatography A*.

[104] Milner J. A. (2001). Mechanisms by which garlic and allyl sulfur compounds suppress carcinogen bioactivation. Garlic and carcinogenesis. *Advances in Experimental Medicine and Biology*.

[105] Ruddock P. S., Liao M., Foster B. C., Lawson L., Arnason J. T., Dillon J.-A. R. (2005). Garlic natural health products exhibit variable constituent levels and antimicrobial activity against Neisseria gonorrhoeae, Staphylococcus aureus and Enterococcus faecalis. *Phytotherapy Research*.

[106] Ross S. A., Milner J., Wildman R. E. C. (2006). Garlic: the mystical food in health promotion. *Handbook of nutraceuticals and functional foods*.

[107] Iciek M., Kwiecien I., Wlodek L. (2009). Biological properties of garlic and garlic-derived organosulfur compounds. *Environmental and Molecular Mutagenesis*.

